# Personalized local SAR prediction for parallel transmit neuroimaging at 7T from a single T1‐weighted dataset

**DOI:** 10.1002/mrm.29215

**Published:** 2022-03-28

**Authors:** Wyger M. Brink, Sahar Yousefi, Prernna Bhatnagar, Rob F. Remis, Marius Staring, Andrew G. Webb

**Affiliations:** ^1^ C.J. Gorter Center for High Field MRI, Department of Radiology Leiden University Medical Center Leiden the Netherlands; ^2^ Division of Image Processing, Department of Radiology Leiden University Medical Center Leiden the Netherlands; ^3^ Circuits and Systems Group, Department of Microelectronics Delft University of Technology Delft the Netherlands

**Keywords:** body models, deep learning, PTx, SAR, subject‐specific

## Abstract

**Purpose:**

Parallel RF transmission (PTx) is one of the key technologies enabling high quality imaging at ultra‐high fields (≥7T). Compliance with regulatory limits on the local specific absorption rate (SAR) typically involves over‐conservative safety margins to account for intersubject variability, which negatively affect the utilization of ultra‐high field MR. In this work, we present a method to generate a subject‐specific body model from a single T1‐weighted dataset for personalized local SAR prediction in PTx neuroimaging at 7T.

**Methods:**

Multi‐contrast data were acquired at 7T (*N* = 10) to establish ground truth segmentations in eight tissue types. A 2.5D convolutional neural network was trained using the T1‐weighted data as input in a leave‐one‐out cross‐validation study. The segmentation accuracy was evaluated through local SAR simulations in a quadrature birdcage as well as a PTx coil model.

**Results:**

The network‐generated segmentations reached Dice coefficients of 86.7% ± 6.7% (mean ± SD) and showed to successfully address the severe intensity bias and contrast variations typical to 7T. Errors in peak local SAR obtained were below 3.0% in the quadrature birdcage. Results obtained in the PTx configuration indicated that a safety margin of 6.3% ensures conservative local SAR estimates in 95% of the random RF shims, compared to an average overestimation of 34% in the generic “one‐size‐fits‐all” approach.

**Conclusion:**

A subject‐specific body model can be automatically generated from a single T1‐weighted dataset by means of deep learning, providing the necessary inputs for accurate and personalized local SAR predictions in PTx neuroimaging at 7T.

## 1 INTRODUCTION

Ultra‐high field MRI (*B*_0_ ≥ 7T) is known to offer higher resolution structural and physiological information than attainable at 3T, particularly in the brain.^1^ At ultra‐high field, parallel transmission (PTx) using multiple RF transmitters is a key technology to address the increased level of non‐uniformity in the RF field distribution.^2^, ^3^, ^4^ PTx allows for dynamic manipulations of the *B*_1_^+^ field distribution by adjusting the RF phases and amplitudes of the individual transmit channels, thereby enabling optimization of the spin excitation process. This flexibility comes at the cost, however, of an increased range of potential local RF power absorption levels in the body, for which in Europe regulatory limits are defined by the IEC in terms of the peak 10 g‐averaged specific absorption rate (SAR).

Although global SAR metrics such as head‐averaged SAR can be adequately monitored via the RF input power, as is commonly done in single‐channel (i.e., non‐PTx) systems, local SAR cannot be measured and is generally a complex function of both system characteristics as well as the subject‐specific anatomy.^5^ Depending on the excitation pattern of the RF transmit array, local SAR can vary by as much as 600% for a given RF input power.^6^ This aspect can be accounted for in the local SAR model by employing the so‐called Q‐matrix formalism,^7^ often compressed to a smaller set of virtual observation points with a pre‐defined safety factor to account for the compression loss.^8^ Additionally, local SAR is known to vary by up to 70% depending on the anatomy of the subject, including aspects such as tissue distribution as well as positioning within the RF coil.^9^, ^10^, ^11^ This intersubject variability is typically estimated offline, by evaluating multiple generic body models, and accompanied with conservative safety margins to ensure compliance in all subjects. This “one‐size‐fits‐all” approach inevitably compromises the RF performance and limits the utilization of PTx at ultra‐high fields, as well as limits our insight into the actual RF exposure levels imposed by ultra‐high field MRI systems.

Several groups have previously demonstrated subject‐specific approaches to SAR prediction by establishing a subject‐specific anatomical model from MR data which is then evaluated in an electromagnetic solver.^12^, ^13^ This builds on the principle that local SAR depends predominantly on the geometry of electrically distinct tissues, rather than their exact dielectric properties.^14^, ^15^ To address the time‐consuming process of image segmentation, techniques based on semi‐automatic segmentation,^12^ image registration,^13^ computer vision^16^, ^17^ and deep learning have been proposed.^18^ The resulting synthesized body model can then facilitate both subject‐specific calculations of local SAR as well as tailored PTx pulse designs, both key to the ultra‐high field MR workflow.^19^, ^20^ As these studies are typically based on 3T data which are relatively free from image artifacts, the resulting image segmentation methods are not directly suited to handle 7T data due to the increased level of image shading and contrast non‐uniformity, which would lead to segmentation errors and inaccuracies in the resulting SAR predictions. Addressing these inaccuracies would require either time‐consuming manual corrections or, alternatively, an additional MR examination at 3T.

In this work, we present a method based on deep learning to generate a subject‐specific numerical body model for local SAR prediction automatically from a single 3D T1‐weighted neuroimaging dataset acquired at 7T, which can be run in a few minutes and is standard in almost all neuroimaging protocols. The network is trained using a custom set of segmented body models derived from multi‐contrast 7T data to serve as the ground truth. By using the original T1‐weighted data as input for training, RF‐induced image nonuniformities and artifacts typical to 7T are automatically accounted for by the network. Finally, the accuracy of the network‐generated body models is evaluated in terms of the 10 g‐averaged SAR in both a quadrature birdcage RF coil model as well as a PTx configuration and compared to the conventional “one‐size‐fits‐all” approach.

## 2 METHODS

**FIGURE 1:**
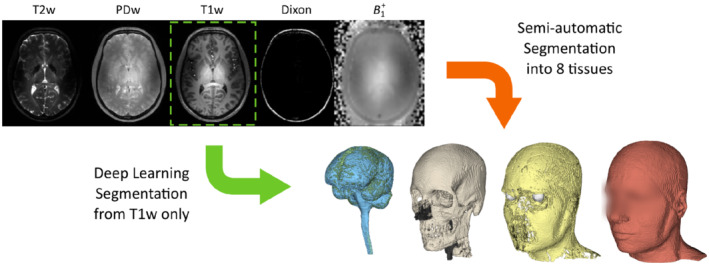
Schematic illustration of the multi‐contrast data used for generating the custom set of body models (*N* = 10) to serve as ground truth, of which the T1‐weighted data is used as input for training the deep learning method. Whereas the semi‐automatic segmentation process involves many steps with elaborate user interaction, the deep learning method produces the body model from the original T1‐weighted data automatically

The approach for developing the custom set of body models and deep learning segmentation method is schematically illustrated in Figure 1 and described in more detailed in the following sections. Healthy volunteers were scanned under a protocol approved by the local institutional review board. Signed informed consent was obtained from all volunteers.

### 2.1 MR protocol

A multi‐contrast MR protocol was acquired in 10 healthy volunteers (5 male, 5 female, age 26.9 ± 9.7) on a 7T MR system (Achieva, Philips Healthcare, Best, the Netherlands) equipped with a quadrature birdcage head coil and a 32‐channel receive coil array (Nova Medical, Wilmington, MA). The imaging protocol started with image‐based *B*_0_ shimming up to third‐order and image‐based receive coil sensitivity calibration in the entire head and neck region using vendor‐supplied routines. All anatomical data were acquired at an isotropic spatial resolution of 1 mm^3^ and a field of view of 192 × 256 × 256 mm^3^ in a sagittal orientation covering the head and neck.

The MR protocol included a T1w 3D MP‐RAGE sequence (TR/TE/TI = 4.9/2.3/1050 ms, shot interval = 2500 ms, 69 shots, flip angle = 5°, sensitivity encoding (SENSE) factor = 1.5 × 2 [AP × RL], acquisition time = 2 min 54 s), a T2w 3D fast spin echo (FSE) sequence (TR/TE/TE_eq_ = 2500/205/132 ms, echo train length (ETL) = 128, refocusing angle = 70°, SENSE factor = 2 × 2, partial Fourier factor = 6/8, number of signal averages = 2, acquisition time = 4 min 5 s), and a PDw 3D spoiled gradient echo sequence (TR/TE = 3.7/1.97 ms, flip angle = 10°, acquisition time = 2 min 39 s). Additionally, a three‐point multi‐acquisition 3D Dixon sequence was acquired for water/fat separation (TR/TE_1_/ΔTE = 6.3/3.0/0.33 ms, flip angle = 15°, SENSE factor = 2 × 2, acquisition time = 5 min 21 s), and *B*_1_^+^ mapping was performed using a multislice DREAM sequence (in‐plane resolution = 4 × 4 mm^2^, slice thickness = 4 mm, TR/TE = 4.0/1.97 ms, STEAM/imaging flip angle = 50°/10°, acquisition time = 13 s).^21^ All image reconstructions were performed twice, with intensity normalization of the receive coils first calibrated to the volume coil and subsequently calibrated to a sum‐of‐squares combination of the receive elements, using vendor‐supplied reconstruction routines. This results in having an intensity bias imprinted on the data that is similar to that obtained either in a transmit/receive RF coil or a receive‐only RF coil array, respectively.

### 2.2 Semi‐automatic segmentation for ground truth generation

The image data were segmented into eight distinct tissue types to ensure accurate predictions of local SAR,^15^ using a semi‐automatic segmentation pipeline involving Matlab 9.10 (MathWorks, Natick, Massachusetts, USA), FSL 6.0 (https://fsl.fmrib.ox.ac.uk/fsl/fslwiki/) and 3D Slicer (https://www.slicer.org/).^22^, ^23^ The target tissue types extended those suggested by the study of Buck et al.^15^ and included internal air, bone, muscle, fat, white matter, gray matter, cerebrospinal fluid, and eye tissue. This resulted in 10 three‐dimensional body models with corresponding T1w image data to serve as pairs of ground truth and input data in the development of the deep learning segmentation method. The approach is graphically illustrated in Figure 1.

**FIGURE 2:**
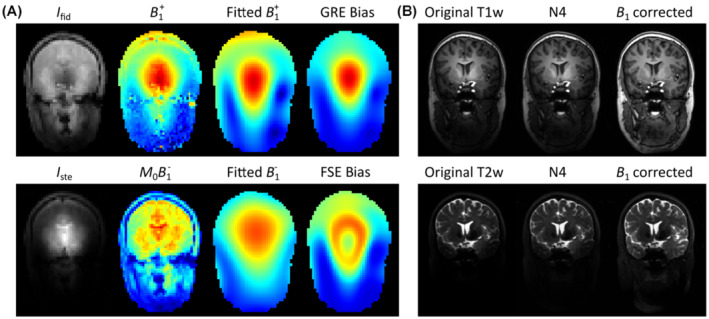
Custom intensity bias correction procedure based on DREAM data. Bias fields for gradient‐recalled (GRE) and fast spin echo (FSE) sequences were estimated by fitting the DREAM‐generated *B*_1_^+^ and *M*_0_ *B*_1_^−^ maps to a spherical function basis (A), which were subsequently used to correct the image data (B)

The semi‐automatic segmentation procedure started with a custom intensity bias correction procedure based on the DREAM data to correct for the RF‐induced nonuniformities in the 7T image data.^24^ The underlying stimulated‐echo and FID images were first used to derive *B*_1_^+^ and *M*_0_ *B*_1_^−^ maps based on the corresponding signal expressions,^21^ which were subsequently fitted onto a spherical function basis to remove the *M*_0_ component and noise.^25^ The fitted maps were then used to generate a bias field estimate by using the signal equations corresponding to gradient‐recalled (GRE) and spin‐echo sequences,^26^ viz.

$${\mathrm{SI}}_{\mathrm{GRE}}=M_{0}\sin\left(\gamma B_{1}^{+}\tau \right)B_{1}^{-}\propto B_{1}^{+}B_{1}^{-}$$

$${\mathrm{SI}}_{\mathrm{FSE}}=M_{0}\sin{\left(\gamma B_{1}^{+}\tau \right)}^{3}B_{1}^{-}\propto \sin{\left(\gamma B_{1}^{+}\tau \right)}^{3}B_{1}^{-}$$

which were applied to the corresponding datasets. The bias correction procedure is graphically illustrated and compared to conventional N4 bias correction in Figure 2. After intensity correction, all datasets were co‐registered using the rigid registration procedure from the Elastix toolbox in 3D Slicer.^27^

Body tissues were distinguished from bone and internal air by thresholding the PDw data, followed by manual correction of image artifacts such as eye motion or residual intensity bias. The PDw data were then median filtered and paranasal sinuses identified within the corresponding cranial bone sections by means of thresholding. Care was taken to ensure that the bone wall around the sinuses was no less than 2 mm thick. Brain extraction and segmentation were performed on the T1w data using the BET and FAST toolboxes within FSL.^28^ The T2w data were used to segment the eyes using a region growing algorithm in 3D Slicer. The remaining body tissues were segmented into fat and muscle based on the fat fraction maps that were derived from the Dixon data. Finally, a 1 mm layer of skin was enforced by replacing fat voxels in the outer layer of the body model with muscle.

### 2.3 Deep learning segmentation

A convolutional neural network was designed based on the ForkNET topology^18^ and implemented using Tensorflow^29^ in Python. The network architecture consists of multiple U‐net structures with one common encoder and nine parallel decoders, each output corresponding to one of the tissue segments in addition to one for the background. As 3D convolutional neural networks often pose demanding memory requirements, a 2.5D approach was adopted by training three independent 2D networks for each of the three orthogonal slice orientations. The network topology had a total of 23 layers, of which 6 were pooling layers. The first layer encoded eight feature maps, and this number doubled after each of the pooling layers. This yielded a total number of 5 million trainable network parameters per 2D network. All convolutions were performed using a kernel size of 3 × 3, stride of 1 × 1 and padding of 1. All deconvolutions and max pooling steps were performed using a kernel size of 2 × 2. Batch normalization was performed with a momentum of 0.9 and a stability parameter of *ε* = 0.001. After summing the three network outputs, tissue labels were assigned according to the maximum output channel. In the case when none of the channels generated an output (i.e., all outputs being equal to zero), which would result in a void voxel within the model, a neighborhood majority vote was applied.^18^

A cross‐validation study was performed to test the performance of the deep learning segmentation method on independent data which were not used for training the network. To achieve this, all training was performed in a leave‐one‐out manner, in which the test subject (i.e., the 3D dataset that was used for testing the network) was excluded from the entire training stage. The network was then trained using randomized 2D slices of the original T1w data (i.e., without any pre‐processing) as input, and corresponding 2D slices of the semi‐automatic segmentations as the ground truth, in which 90% of the dataset was used for training and 10% for validation. This means that the transverse and coronal networks were trained with 2304 slices of 192 × 256 pixels in size and that the sagittal network was trained with 1728 slices of 256 × 256 pixels in size. Either the T1w data with volume coil or sum‐of‐squares intensity normalization were used as input data, yielding a dedicated network for either reconstruction setting. Training was performed using batches of 10 randomized training images per iteration in 40 epochs using the ADAM optimizer.^30^ The Dice coefficient, also known as Dice similarity index, was used to measure segmentation quality and employed as a loss function for training. One epoch took approximately 114 s on a GPU (Tesla K40c, NVIDIA, Santa Clara, CA), which resulted in a total training time of approximately 4 h per test subject. After training and testing, the network was re‐initialized with random weights, and the procedure was repeated on the following test subject such that the accuracy of the method could be evaluated in all datasets (*N* = 10) in an independent manner.

### 2.4 RF field simulations

RF field simulations on the ground truth and network‐generated body models were obtained at 300 MHz using XFdtd (version 7.4, Remcom Inc., State College, PA) to evaluate the *B*_1_^+^ and 10 g‐averaged SAR distribution (SAR_10g_). Literature values for the dielectric properties and density were assigned to each of the tissue types.^31^ SAR averaging was performed using a custom region growing algorithm, which ensures correct averaging around the outer borders of the model.^32^ All simulations were performed in a 2 mm uniform discretization grid with a sinusoidal excitation at 300 MHz on an Intel Xeon 2.80 GHz processor equipped with a GPU (Tesla K40c, NVIDIA, Santa Clara, CA), and all custom post‐processing was implemented in Matlab (version 9.10, MathWorks, Natick, MA).

First, a single‐channel RF exposure assessment was performed on each of the body models in a shielded 16‐rung high‐pass birdcage model driven in quadrature mode using fixed excitation ports at each of the capacitor gaps. The rungs of the birdcage were 18 cm long and 2.5 cm wide, the inner diameter was 30 cm and the outer diameter of the shield was 36 cm. The birdcage RF coil model was validated experimentally in a head‐sized phantom through *B*_1_^+^ mapping as well as MR thermometry.^33^ Simulations in the birdcage model took approximately 130 s to reach a steady state with −40 dB of convergence, owing to the non‐resonant nature of the coil model, and the resulting field data were normalized to 1 W of RF input power.

A PTx RF exposure assessment was finally carried out on each of the body models by evaluating 1000 random RF shims in a generic eight‐channel unshielded loop array coil with an inner diameter of 30 cm. The loop elements had a 6 cm width and 24 cm length and had six tuning capacitor breaks. The RF coil was simulated using excitation ports at each of the 48 capacitor gaps and tuned using a circuit co‐simulation method which involved a custom optimization procedure aimed to minimize both the input reflection coefficients and worst case coupling between channels.^34^, ^35^ The tuning process was performed by loading the coil with a reference body model “Duke” from the Virtual Family,^36^ and yielded tuning capacitances of 3.6 pF and a series matching capacitor of 5.9 pF. All input reflection coefficients were below −12 dB, hence the coil model did not require retuning when different body models were inserted. After tuning the coil in the circuit co‐simulation domain, field data were combined to produce the *B*_1_^+^ and electric field response for each of the channels. The electric field data were then combined to construct Q‐matrices,^37^ which were averaged over 10 g of tissue and converted into a vectorized format to allow for efficient evaluation of the local SAR in arbitrary RF shim settings.^19^, ^20^ A series of 1000 random RF shims was finally evaluated in both the ground truth as well as the network‐generated body models by assigning random phases and amplitudes to all RF channels and comparing the resulting SAR_10g_ distributions. All PTx simulation results were normalized to a total input power of 1 W. Port‐wise simulations of the PTx coil model took around 30 s per port and post‐processing (i.e., circuit co‐simulation and averaging of the Q‐matrices) took around 100 s. In all, the PTx exposure analysis in a single body model took approximately 25 min.

## 3 RESULTS

### 3.1 Deep learning segmentation

**FIGURE 3:**
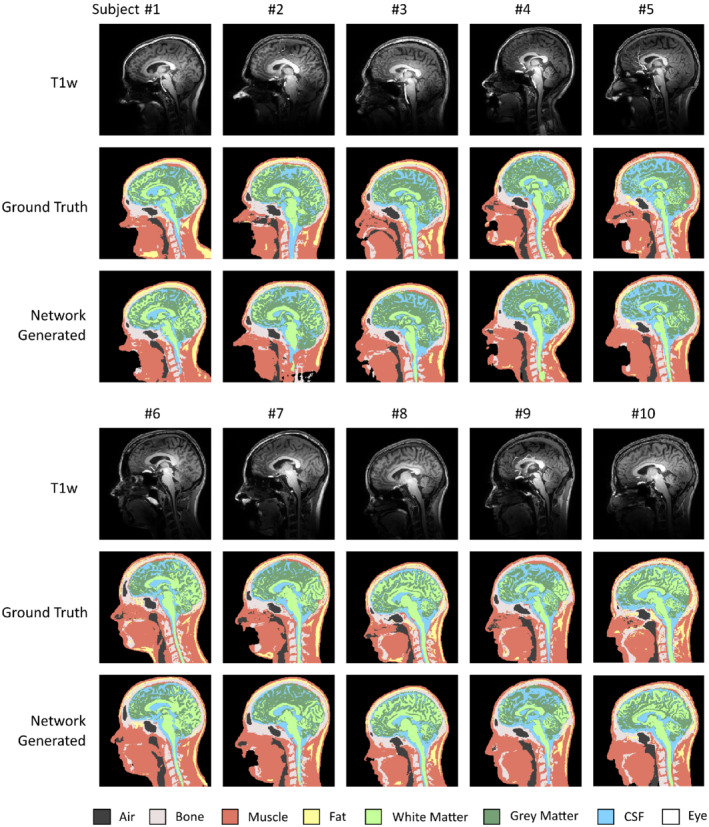
Leave‐one‐out cross‐validation results comparing ground truth and deep learning‐based segmentations in all volunteers. Shown are sagittal cross‐sections of the T1‐weighted data (top), ground truth segmentations (middle), and network‐generated segmentations (bottom). The deep learning method shows to account for the nonuniform contrast and severe drop‐off in intensity towards the neck. In each of these evaluations, the test subject was excluded from the training data to ensure generalizability

The segmentation results of the leave‐one‐out cross‐validation study are shown in Figure 3. The network‐generated models showed a strong similarity with the ground truth models, indicating that the network was able to account for the non‐uniform intensity and contrast variations within the head as well as the strong signal drop‐off towards the neck. In particular, the paranasal sinuses and bone segments were correctly distinguished despite having a very similar signal intensity in the T1w data, indicating the leverage obtained through the deep learning approach. Some models showed some undersegmentation in distal neck regions where SNR was very low, however this may not be problematic as local SAR is typically low here as well. On average, around 158 voxels within the 3D model were not classified by any of the decoder branches and were generated using the neighborhood majority vote rule. Results obtained for the sum‐of‐squares intensity normalized data were essentially the same.

**FIGURE 4:**
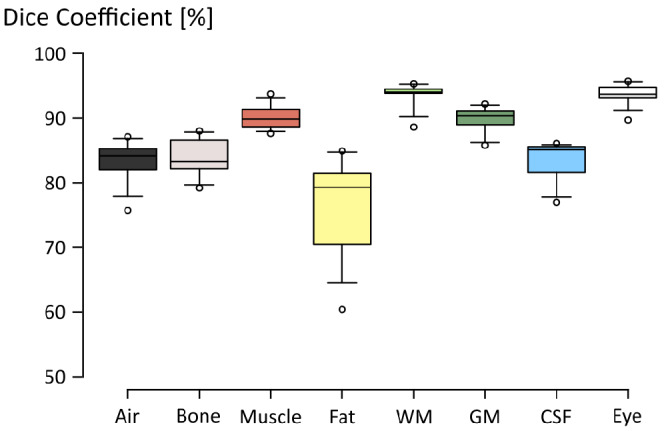
Boxplot diagram showing the Dice coefficients obtained in the cross‐validation study (*N* = 10). Center lines indicate median values, box limits indicate 25th and 75th percentiles, whiskers extend to 5th and 95th percentiles and outliers are represented by dots

The Dice coefficients for the different tissue segments in the cross‐validation study are shown in Figure 4, showing an overall Dice coefficient of 86.7% ± 6.7% (mean ± SD). Median Dice coefficients were greater than 80% in all segments, with fat reaching the lowest overall accuracy. We note that this metric reflects segmentation errors in the entire field of view, including areas where the SAR_10g_ is typically low, for example in the neck where the gross anatomy is expected to be more relevant than the local tissue properties. Structures with a well‐defined MR contrast and shape, such as white matter and eye tissues, reached the highest overall dice coefficients.

### 3.2 RF field simulations

**FIGURE 5:**
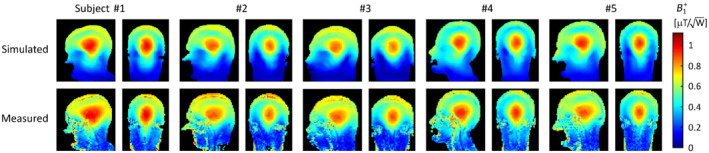
Experimental validation of the ground truth segmentations in the quadrature birdcage RF coil. Shown are the simulated (top) and measured (bottom) *B*_1_^+^ data. All data were normalized to 1 W of input power

The accuracy of the network‐generated body models was evaluated by comparing simulations and measurements of the *B*_1_^+^ field in the quadrature birdcage RF coil model, which are shown in Figure 5. The simulated *B*_1_^+^ shows a high degree of correspondence with the measured *B*_1_^+^ data, both in terms of the relative distribution as well as in terms of peak transmit efficiency.

**FIGURE 6:**
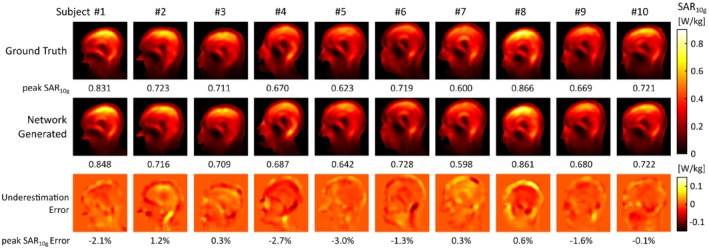
Quadrature birdcage local SAR assessment. Shown are simulated SAR_10g_ distributions in ground truth (top) and network‐generated body models (middle), and corresponding underestimation error maps (bottom). Figure footers denote peak SAR_10g_ values (top, middle) and the corresponding relative underestimation (bottom). Positive errors indicate a peak SAR_10g_ underestimation in the network‐generated model

Simulations of the SAR_10g_ distribution in the ground truth and network‐generated body models obtained in the quadrature birdcage model are shown in Figure 6. The bottom row shows the voxel‐wise underestimation error obtained by subtracting the SAR_10g_ data obtained in the network‐generated model from those obtained in the ground truth model. In other words, underestimation of SAR_10g_ (i.e., undesired from a safety compliance point of view) corresponds to a positive underestimation error. The peak SAR_10g_ values obtained in the network‐generated body models were within 3.0% of those obtained in the corresponding ground truth body models, for all subjects. This is considerably lower than the intersubject variability in peak SAR_10g_ of 37.2% (i.e., absolute range divided by the mean value) and practical uncertainty levels associated with RF exposure assessments.^6^, ^15^, ^33^ The head‐averaged SAR values obtained in the network‐generated models were within 1.8% of those obtained in the ground‐truth models.

**FIGURE 7:**
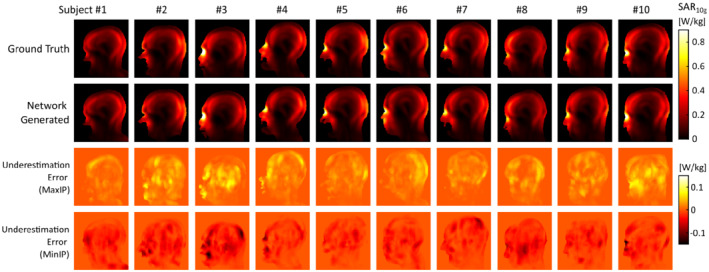
PTx local SAR assessment. Shown are maps of the maximum SAR_10g_ value obtained in the evaluation of 1000 random RF shims in the ground truth (top) as well as network‐generated models (middle), and projections of the SAR_10g_ underestimation and overestimation (bottom). Positive errors indicate SAR_10g_ underestimation in the network‐generated model

Results of the PTx RF exposure assessment are shown in Figure 7, showing sagittal cross‐sections of the maximum SAR_10g_ value obtained in the 1000 random RF shims. Both maximum as well as minimum intensity projections of the voxel‐wise underestimation error are shown in the two bottom rows, where the underestimation error corresponds to the maximum SAR_10g_ maps, here.

**FIGURE 8:**
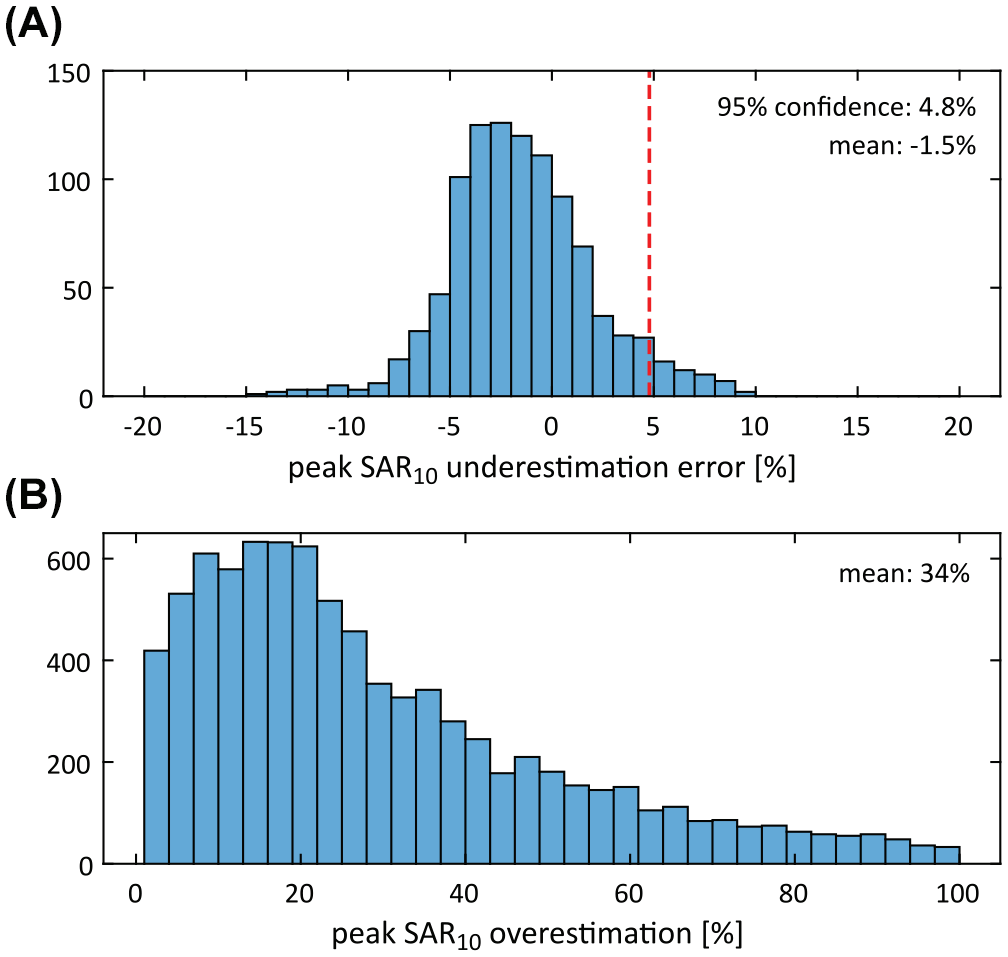
Statistical analysis of the peak SAR_10g_ accuracy obtained with the deep learning segmentation method in the PTx configuration in 1000 random PTx excitation settings. Shown are histograms of the peak SAR_10g_ underestimation error obtained in the network‐generated models (A) and the peak SAR_10g_ overestimation obtained in the generic “one‐size‐fits‐all” approach (B)

An overview of the peak SAR_10g_ underestimation error in the PTx configuration is shown in Figure 8a, obtained by comparing the peak SAR_10g_ produced in each of the network‐generated models with that produced in the corresponding ground truth model, for each of the 1000 random RF shims. Figure 8B shows the peak SAR_10g_ overestimation error in the generic “one‐size‐fits‐all” approach, obtained by comparing the peak SAR_10g_ produced in each of the ground truth body models with the maximum peak SAR_10g_ that is produced in the other nine body models of the dataset, for each of the 1000 random RF shims. The underestimation error had a mean value of −1.5%, which corresponds to a slight overestimation of the peak SAR_10g_, and in 95% of the RF shims the underestimation error was found to be less than 4.8%. By incorporating these into a safety factor, the subject‐specific approach would incur an effective peak SAR_10g_ overestimation of up to 6.3% with a 5% probability of underestimation, whereas the generic approach would result in an average overestimation of 34%, reaching over 95% of overestimation in 5% of the cases. For comparison, increasing the confidence interval of the safety factor to 99% would lead to an effective peak SAR_10g_ overestimation of up to 9% with a 1% probability of underestimation.

## 4 DISCUSSION

In this work we have explored the potential of deep learning for generating a subject‐specific numerical body model from a single T1‐weighted 7T image dataset for personalized local SAR prediction. Local SAR compliance is one of the current bottlenecks hindering clinical use of PTx at 7T. Most vendors impose restrictive safety margins on the use of PTx of up to 300% to ensure compliance, which compromise image quality by limiting the allowed range of sequence parameters such as the refocusing tip angles in FSE sequences or the minimum repetition time that can be attained. Such compromises make that 7T is currently not utilized to its full potential, limiting its clinical impact. Subject‐specific information on local SAR would enable tailoring the RF safety margins to the individual subject, rather than applying generic models with overconservative safety margins, thereby removing unnecessary limitations and enabling PTx to be exploited at its full potential.

The segmentation performance of the proposed deep learning approach was found to be of high quality, as reflected in the local SAR results. By training the network on 7T MR data with severe intensity bias and contrast non‐uniformities throughout the field of view, the method was found to correctly account for these intrinsic image characteristics, despite that only nine subjects were used for training the network in each of the cross‐validation cycles. This means that the method relieves the operator from performing elaborate bias‐correction procedures or other image processing steps, but instead can be directly applied to the 7T data without any pre‐processing. Of all tissues, fat reached the lowest overall segmentation accuracy with a median Dice coefficient of 80%. This can be explained by the different MR contrast mechanisms that were used, with ground truth segmentations being based on chemical shift, encoded in the Dixon data, as opposed to the T1‐weighted contrast of the input data. From Figure 3, it can be observed that fat is often undersegmented in the lower portion of the body models. This also corresponds to the region where the adiabatic RF inversion pulse fails to reach a proper inversion, explaining the inconsistent T1‐weighting of the input data in this region. Other groups have proposed acquiring multiple MR contrasts or even MR fingerprinting as input data to improve the segmentation quality^17^, ^38^; however, such approaches would substantially increase the acquisition time and interfere with the MR workflow. Finally, the ForkNET network design was chosen here, and was previously shown by Rashed et al. to outperform a conventional U‐NET in semantic segmentation of MRI data; however, other network designs may also be conceivable. This may also involve different loss functions, such as cross entropy, or include attention mechanisms to promote SAR‐sensitive regions of the model to be represented with improved quality.^39^

In the current study, the RF exposure assessment took approximately 2 min in the quadrature birdcage model and 25 min. in the PTx configuration, both relatively time‐consuming compared to the deep learning segmentation step taking only 14 s. Together with the acquisition of the T1w input data, which took almost 3 min, this constitutes a total workflow of around 6 min for the single‐channel RF exposure assessment and close to 30 min for the PTx exposure analysis. Future work should therefore aim to reduce both the MR data acquisition and RF simulation time, to improve the integration of the subject‐specific approach into the MR workflow. Options to speed up the RF simulations would include using a larger simulation grid size, leveraging parallel computing as well as using specialized EM solvers such as MARIE.^40^ For example, increasing the simulation grid from 2 mm to 4 mm reduces the computation time for the PTx exposure analysis from 25 min to around 7 min. In a PTx setting, we should note that the *B*_1_^+^ predictions obtained from the RF simulations would also allow subsequent PTx pulse calibrations, potentially saving time by avoiding volumetric *B*_1_^+^ mapping procedures, which can take several minutes to acquire.^41^, ^42^, ^43^

Recently, other groups explored methodologies to infer local SAR directly from *B*_1_^+^ maps using deep learning,^44^ exploiting the coupled structure of the magnetic and electric RF fields, or even directly from anatomical MR images.^45^ Although such approaches show potential to resolve local SAR in a single‐channel configuration or for a specific RF shim setting, these have not yet been demonstrated in a comprehensive PTx workflow, which would require channel‐wise local SAR information as well as information accounting for the interference between the different channels. Our approach has the advantage that the subject‐specific anatomical model can be used to perform a full RF exposure analysis, including for example channel‐wise analyses or dedicated PTx excitation settings. Additionally, our approach can potentially handle MRI data from a wider variety of RF coils, as most PTx arrays optimized for neuroimaging are capable of generating a circular polarized (CP_1_^+^) mode that will produce an excitation *B*_1_^+^ field very similar in distribution to that obtained in the quadrature birdcage, which was used here. This would then also produce contrast variations and intensity bias effects comparable to those present in the data used for training the network. Additionally, different receive channel combination strategies have been addressed by including both sum‐of‐squares as well as volume‐coil normalized data in the training dataset. Remaining intersystem variations in image intensity are anticipated to fall well within the range of intersubject variations, which the network was well capable of addressing as shown by the current study.

Limitations of the current study include the limited size of the dataset (*N* = 10). In a previous segmentation study at 3T stable training was obtained with a similar number of subjects.^46^ To determine whether this was also adequate in the current study, we evaluated the convergence of the leave‐one‐out cross‐validation study when using fewer subjects, for example, *N* = 5 up to *N* = 10 (cf. Supporting Information Figure S1, which is available online). The peak SAR_10g_ error was found to be no greater than 3.1% and converged smoothly to the values obtained when all subjects were included. Although this suggests generalizability of the network, segmentations in subjects with a significantly different anatomy, for example, pediatric subjects or in specific pathologies, may potentially reveal inconsistencies and may require further extensions of the training dataset. A challenge with including pathologies in the training data is that it is not yet clear whether the dielectric properties could still be represented using the current set of tissue clusters. Another limitation of our study, and of RF exposure assessments in general, is that it is not possible to validate the RF simulation results with in vivo measurements of the SAR distribution. We have experimentally validated our head models by comparing the measured and simulated *B*_1_^+^ fields in the birdcage model, which despite showing a strong agreement leave some room for further model refinements. An underlying shortcoming in this validation approach, is that errors in local SAR may not always be directly reflected in the *B*_1_^+^ distribution.^47^ Additionally, the PTx exposure analysis was only performed in a single PTx coil model, and other PTx coil designs may show a different sensitivity to segmentation errors. Finally, in the PTx analysis, we considered only static RF shimming with random excitation settings, which also includes settings that do not produce practically useful *B*_1_^+^ distributions. Although this enables generalization of the results, a more realistic analysis could target tailored PTx pulses such as kT‐points, SPINS pulses or local SAR‐optimized RF pulse designs, specifically.^19^, ^20^, ^48^, ^49^

## 5 CONCLUSIONS

In this work, we demonstrate a method based on deep learning to automatically generate a subject‐specific numerical body model from a single T1‐weighted 7T image dataset for personalized RF exposure prediction. The network‐generated body models showed reproduction of the ground truth RF exposure results with a high level of agreement, with peak local SAR errors below 3.0% in the quadrature birdcage model. In the PTx configuration, a safety margin of 6.3% was sufficient to ensure a conservative local SAR prediction in 95% of the random RF shims, compared to an average overestimation of 34% in the “one‐size‐fits‐all” approach. As a T1‐weighted image is typically acquired at the start of a neuroimaging protocol as a basic anatomical reference, the procedure has the potential to be seamlessly integrated into the MR workflow.

## Supporting information


**Figure S1.** Convergence of the leave‐one‐out cross‐validation study evaluated in the quadrature birdcage configuration. When using fewer subjects (*N* = 5) the peak local SAR_10g_ is within 3.1% compared to the cross‐validation result based on using all subjects (*N* = 10). Values shown are peak SAR_10g_ (top) and relative peak SAR_10g_ error (bottom) compared to the value obtained when using all subjects (*N* = 10).

## Data Availability

Python source code and trained networks are available for download via https://github.com/wygerbrink/PersonalizedDosimetry.
