# Supplementary figures and images for: Personalized local SAR prediction for parallel transmit neuroimaging at 7T from a single T1‐weighted dataset
Source: Magn Reson Med. 2022 Mar 28;88(1):464–75. doi: 10.1002/mrm.29215 (PMC9314883; doi:10.1002/mrm.29215)

# Supporting Information Figure S1

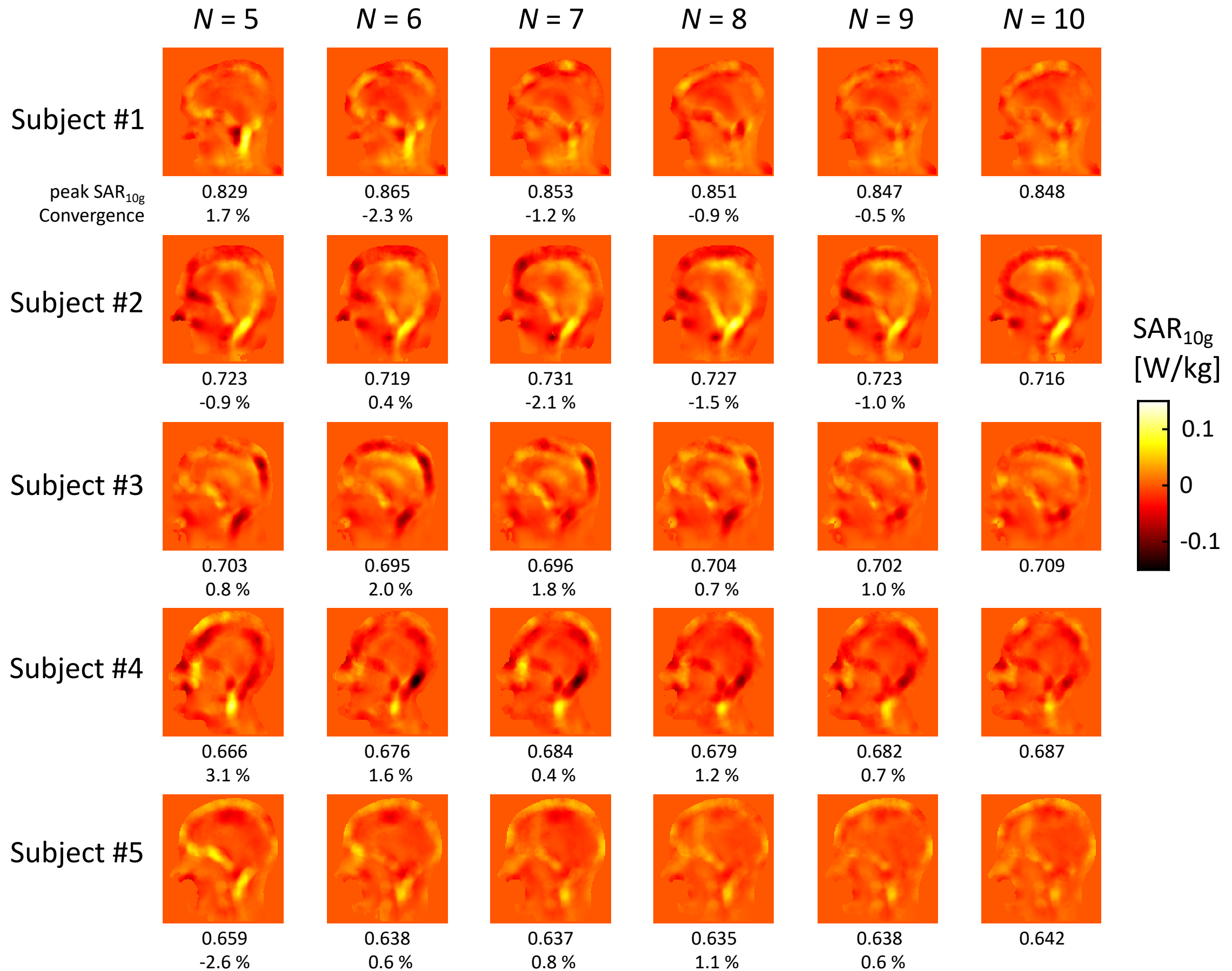

Supplement: Supplementary file 1 — Figure S1. Convergence of the leave‐one‐out cross‐validation study evaluated in the quadrature birdcage configuration. When using fewer subjects (N = 5) the peak local SAR10g is within 3.1% compared to the cross‐validation result based on using all subjects (N = 10). Values shown are peak SAR10g (top) and relative peak SAR10g error (bottom) compared to the value obtained when using all subjects (N = 10). [file MRM-88-464-s001.pdf]
